# The Application and Comparison of Machine Learning Models for the Prediction of Breast Cancer Prognosis: Retrospective Cohort Study

**DOI:** 10.2196/33440

**Published:** 2022-02-18

**Authors:** Jialong Xiao, Miao Mo, Zezhou Wang, Changming Zhou, Jie Shen, Jing Yuan, Yulian He, Ying Zheng

**Affiliations:** 1 Department of Epidemiology School of Public Health Fudan University Shanghai China; 2 Department of Cancer Prevention Fudan University Shanghai Cancer Center Shanghai China; 3 Department of Oncology Shanghai Medical College Fudan University Shanghai China; 4 Shanghai Engineering Research Center of Artificial Intelligence Technology for Tumor Diseases Shanghai China

**Keywords:** breast cancer, machine learning, survival analysis, random survival forest, support vector machine, medical informatics, prediction models

## Abstract

**Background:**

Over the recent years, machine learning methods have been increasingly explored in cancer prognosis because of the appearance of improved machine learning algorithms. These algorithms can use censored data for modeling, such as support vector machines for survival analysis and random survival forest (RSF). However, it is still debated whether traditional (Cox proportional hazard regression) or machine learning-based prognostic models have better predictive performance.

**Objective:**

This study aimed to compare the performance of breast cancer prognostic prediction models based on machine learning and Cox regression.

**Methods:**

This retrospective cohort study included all patients diagnosed with breast cancer and subsequently hospitalized in Fudan University Shanghai Cancer Center between January 1, 2008, and December 31, 2016. After all exclusions, a total of 22,176 cases with 21 features were eligible for model development. The data set was randomly split into a training set (15,523 cases, 70%) and a test set (6653 cases, 30%) for developing 4 models and predicting the overall survival of patients diagnosed with breast cancer. The discriminative ability of models was evaluated by the concordance index (C-index), the time-dependent area under the curve, and D-index; the calibration ability of models was evaluated by the Brier score.

**Results:**

The RSF model revealed the best discriminative performance among the 4 models with 3-year, 5-year, and 10-year time-dependent area under the curve of 0.857, 0.838, and 0.781, a D-index of 7.643 (95% CI 6.542, 8.930) and a C-index of 0.827 (95% CI 0.809, 0.845). The statistical difference of the C-index was tested, and the RSF model significantly outperformed the Cox-EN (elastic net) model (C-index 0.816, 95% CI 0.796, 0.836; *P*=.01), the Cox model (C-index 0.814, 95% CI 0.794, 0.835; *P*=.003), and the support vector machine model (C-index 0.812, 95% CI 0.793, 0.832; *P*<.001). The 4 models’ 3-year, 5-year, and 10-year Brier scores were very close, ranging from 0.027 to 0.094 and less than 0.1, which meant all models had good calibration. In the context of feature importance, elastic net and RSF both indicated that TNM staging, neoadjuvant therapy, number of lymph node metastases, age, and tumor diameter were the top 5 important features for predicting the prognosis of breast cancer. A final online tool was developed to predict the overall survival of patients with breast cancer.

**Conclusions:**

The RSF model slightly outperformed the other models on discriminative ability, revealing the potential of the RSF method as an effective approach to building prognostic prediction models in the context of survival analysis.

## Introduction

Breast cancer is a leading cause of morbidity and mortality in women worldwide, and the prediction of breast cancer prognosis is crucial for decision-making. Accurate outcome prediction can assist doctors with providing appropriate treatment plans for patients, which in turn could improve their chances of survival and lessen the suffering. Several prognostic prediction models have already been developed. PREDICT and Adjuvant! Online are 2 famous prognostic prediction tools for breast cancer based on clinical and pathological characteristics [1,2]. These models have been validated by external data set and are commonly used in the United States and Western Europe. However, several external validations that were made in Asian countries revealed a less-than-optimal predictive ability [3-6].

For survival analysis of follow-up observations, the most important challenge is dealing with censored data. The Cox proportional hazard regression is a classical modeling method used to analyze right-censored data in survival analysis with good interpretability. Typically, the Cox proportional hazard regression imposes proportional hazard assumption and the assumption that continuous covariates have a linear effect on the logarithm of the hazard, which the real-world data may not satisfy [7]. Compared with the Cox proportional hazard regression, machine learning methods do not make any parametric or semiparametric assumptions and have the ability to detect and account for higher-order interactions as well as nonlinear relationships [8]. While there have been some attempts to use machine learning to build cancer prognosis prediction models [6,9-13], currently, there is no consensus on whether traditional or machine learning-based prognostic prediction models have a better predictive performance.

Here, we discuss two main types of prognostic prediction models using machine learning algorithms. The first types are the binary classification models, which give a probability of the interested outcome at a specific time. Several studies have used machine learning methods to generate prognostic prediction models based on classification. The outcome variable of these models is the status of survival at 5 years [14-17] or at the time of data collection [18,19]. The limitation of these models is that they are not able to include right-censored observations that were censored before the specified time, because the outcome of these observations is unknown. Moreover, using the classification outcome (survival status at a specific time) instead of the survival outcome (survival time and status of the censor) can lead to a loss of information. The second types are models using improved algorithms of original machine learning algorithms to enable modeling and analysis of censored data, such as support vector machines (SVM) for survival analysis [20] and random survival forest (RSF) [21]. These methods can describe probability (RSF) and risk scores (SVM and RSF) of the interested outcomes at different time points rather than at a specific time point and can consider both the survival time and the status of the censor.

In this study, traditional (Cox) and machine learning-based (SVM and RSF) prognostic prediction models were developed for patients with breast cancer based on a large cohort of Chinese patients diagnosed with breast cancer and hospitalized in Fudan University Shanghai Cancer Center. We aimed to compare the performance of different models to pick the optimal predictive model and provide a reference for the development of machine learning in the prognosis prediction of breast cancer.

## Methods

### Study Design and Ethical Considerations

This retrospective cohort study included all patients diagnosed with breast cancer and subsequently hospitalized in Fudan University Shanghai Cancer Center between January 1, 2008, and December 31, 2016. Data containing demographic and clinicopathologic features were obtained from the hospital information system. Overall Survival, defined as the duration between the time of first treatment and the date of death, was taken as the outcome to build the predictive models. The outcome information was derived from medical visit records, telephone visits, and death certificate data linkage with the cancer registry system or death certificate system run by the provincial Centers for Disease Control and Prevention.

By March 1, 2021, medical information and follow-up information were collected from 25,629 patients. After excluding male patients, patients with bilateral breast cancer (362 cases), and patients with ≥3 missing features, 22,176 cases with 21 features were eligible for further analysis. Patients were followed for a median follow-up time of 68.9 months (95% CI 68.42, 69.33). The data set was then randomly split into a training set (15,523 cases, 70%) and a test set (6653 cases, 30%). The statistical description of features and the survival curves of patients in the training and test set are shown in Table S1 and Figure S1 in Multimedia Appendix 1.

This study was approved by the Fudan University Shanghai Cancer Center Institutional Review Board (Registration YF-2021-01).

### Preprocess of Missing Data

Since the data were generated and collected in a real medical environment, there were many observations with missing features. As the SVM and RSF methods do not support the analysis of data sets with missing values, we performed a 2-step process in order to reduce the impact of missing values on the training process of developing prediction models. Firstly, we excluded patients with too many missing features. The number of missing features of patients and the log-rank test results are shown in Table S1 in Multimedia Appendix 2. The log-rank method was used to test the difference between the survival state of 25,267 patients and the remaining patients. Based on the results of the log-rank test, when we excluded patients with ≥3 missing features, there was no significant difference between the survival of the remaining patients (22,176 cases) and the survival of the overall patients (25,267 cases; *P*=.17). Therefore, 3 was taken as the cut-off value, and patients with ≥3 missing features were excluded. The statistics for missing features before and after the first step of processing are shown in Table S2 in Multimedia Appendix 2, and the remaining 22,176 cases are eligible for further analysis. Secondly, the remaining missing data were imputed by the missForest algorithm using library “missingpy” (0.2.0) in Python (Python Software Foundation). MissForest is a nonparametric imputation method that could be applied for both continuous and categorical variables and does not make explicit assumptions about the functional form of the data [22]. In the process of imputing the missing values, the outcome data were not involved in case imputed data were affected and falsely related to the outcome data.

### Statistical Analysis

The objective outcome in the study was time to event, which is right-censored survival data. Therefore, the following 3 survival modeling approaches were used to predict the survival time of patients diagnosed with breast cancer: Cox proportional hazard regression [23], SVM [24], and RSF [21]. Elastic Net (EN) was used as the feature selection method to screen important features to train the 3 models. Technical implementation details, including the libraries and the process of hyperparameter tuning, are provided in the Multimedia Appendix 3. Moreover, we have open sourced the Python and R code that we developed for generating the models and evaluating the performance of the models in the GitHub repository [25].

The Cox proportional hazard regression is a classical modeling method for survival analysis. The model predicts the probability that the event of interest has occurred at a given time for given values of the predictor variables [23]. We added a traditional feature selection method for the Cox model, where univariate Cox analysis was performed before significant (*P*<.1) and clinically relevant features were forced into multivariate Cox regression analysis. The Cox model using the EN method was named “Cox-EN,” and the one using the traditional variable selection method was named “Cox.”

Usually, the predictors should satisfy the proportional hazard assumption in the Cox model. However, the main goal of modeling in this study was survival prediction and maximizing concordance index (C-index) and time-dependent area under the curve (AUC), regardless of how predictions are generated. Therefore, we did not perform the test for proportional hazards in the process of modeling [26].

SVM is a supervised machine learning algorithm, which can be used for both classification and regression challenges. An SVM model is basically a representation of different classes in a hyperplane in multidimensional space. The hyperplane is generated iteratively by SVM so that the error can be minimized. The goal of SVM is to divide the data sets into classes to find a maximum marginal hyperplane [24].

Several extensions of SVM to survival analysis were proposed. Shivaswamy et al [27] introduced an approach for censored targets by casting survival analysis as a regression problem. Van Belle et al [24,28] proposed the ranking approach and the hybrid approach combining the regression and ranking approach for survival outcomes. As an objective function of the ranking-based technique depends on a quadratic number of constraints with respect to the number of training samples, which makes training intractable with medium to large-sized data sets, we chose an approach of efficient training of linear survival SVM [20].

RSF, which was developed by Ishwaran et al [21], is an ensemble of tree-based learners for survival analysis of right-censored data and an extension of the random forest method. Using independent bootstrap samples, each tree in RSF is grown by randomly selecting a subset of features for each node and then splitting the node using a survival criterion involving information of survival time and censoring status [21].

EN is a feature screening technique that uses the penalties L1 and L2 from both the least absolute shrinkage and selection operator (LASSO) and ridge techniques to regularize regression models. The EN method is improved based on the shortcomings of both ridge and LASSO methods. The ridge method keeps all the features and cannot perform the function of feature screening. When it comes to multiple correlated features, the LASSO method randomly picks one of these features from such groups and entirely ignores the rest, while the EN method is likely to pick a few at once [29].

### Evaluation of Model Performance

The discriminative ability of models was evaluated by the C-index [30], time-dependent AUC [31], and D-index [32]. C-index measures the overall discriminative ability of models, while time-dependent AUC measures the discriminative ability of models by comparing the predicted probabilities with the actual binary survival status and the probability estimation of a death outcome of censored observations at an interested time. C-index and time-dependent AUC both range in an interval from 0 to 1, and a value of 0.5 is comparable to random guessing, while a value of 1 means perfect discrimination. D-index was used to measure the separation between patients from equally sized high-risk and low-risk groups divided according to the risk score obtained from different models. Higher values of D-index indicate a more remarkable discriminative ability of the model. The survival curves of high-risk and low-risk groups was estimated using the Kaplan-Meier method, and the log-rank test was used to compare survival curves. The calibration ability of models was evaluated by the Brier score [33], which varies between 0 and 1, while a lower Brier score was indicative of a better-calibrated prediction. A value of 0.25 is comparable to random guessing, while a value of 0 means perfect discrimination.

## Results

### User and Model Statistics

A total of 22,176 patients with 68.9 months (95% CI 68.42, 69.33) of median follow-up were included in this study. We fitted 4 prognostic models (Cox, Cox-EN, RSF, and SVM) for predicting the overall survival of breast cancer patients with the training set and then used C-index, time-dependent AUC, D-index, and Brier score to evaluate them in the independent test set. All models showed good calibration, and RSF outperformed other models on discriminative ability with a C-index of 0.827 (95% CI 0.809, 0.845).

### Evaluation of Feature Importance

**Figure 1:**
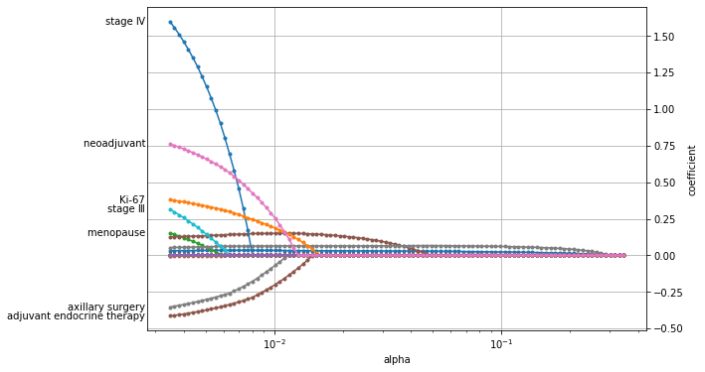
The coefficients of features change for varying α.

**Figure 2:**
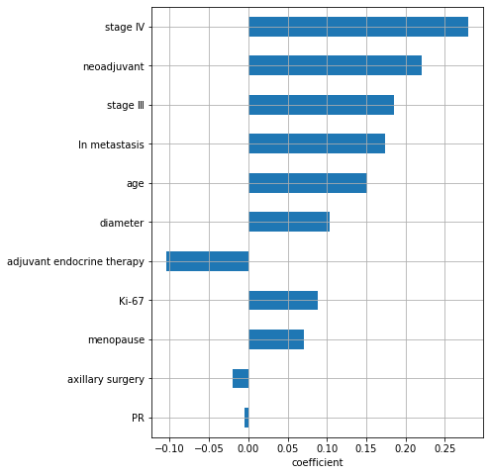
The important coefficient of each feature corresponding to the optimal α by elastic net. Ln: lymph node; PR: progesterone receptors.

**Figure 3:**
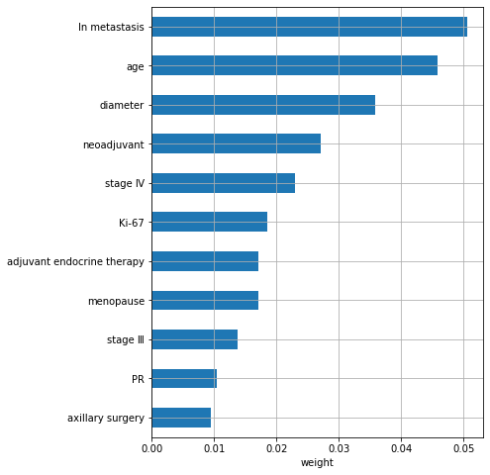
The important coefficient of each feature by random survival forest. Ln: lymph node; PR: progesterone receptors.

In order to screen out features with a large contribution to predicting the prognosis of breast cancer, the EN was first used to select important features, resulting in a total of 21 features. The ways the coefficients changed for varying α is shown in Figure 1, and the coefficient of each feature corresponding to the optimal α is shown in Figure 2. The top 5 important features were TNM staging, neoadjuvant therapy, number of lymph node metastases, age, and diameter of the tumor. RSF was used to rank the importance of the 11 features selected by the EN, and the results are shown in Figure 3. The top 5 important features were the number of lymph node metastasis, age, tumor diameter, neoadjuvant therapy, and TNM staging.

The results of univariate and multivariate Cox analysis are shown in Multimedia Appendix 4. Except for cases of the side of the tumor, multiple tumors, adjuvant chemotherapy, and targeted therapy, all features had a *P* value of less than .1 in the univariate analysis. Considering that adjuvant chemotherapy and targeted therapy could be confounding factors, multivariate analysis was performed using adjuvant chemotherapy, targeted therapy, and the significant factors (*P*<.1) from univariate analysis. The results of the multivariate analyses showed that age, menopause, invasive, diameter, lymph node metastasis, TNM, Ki 67, estrogen receptors, progesterone receptors, breast surgery, axillary surgery, adjuvant chemotherapy, targeted therapy, adjuvant radiotherapy, adjuvant endocrine therapy, and neoadjuvant therapy had a *P* value of less than .05, and the Cox model was built by these features.

### Methods Performance

**Figure 4:**
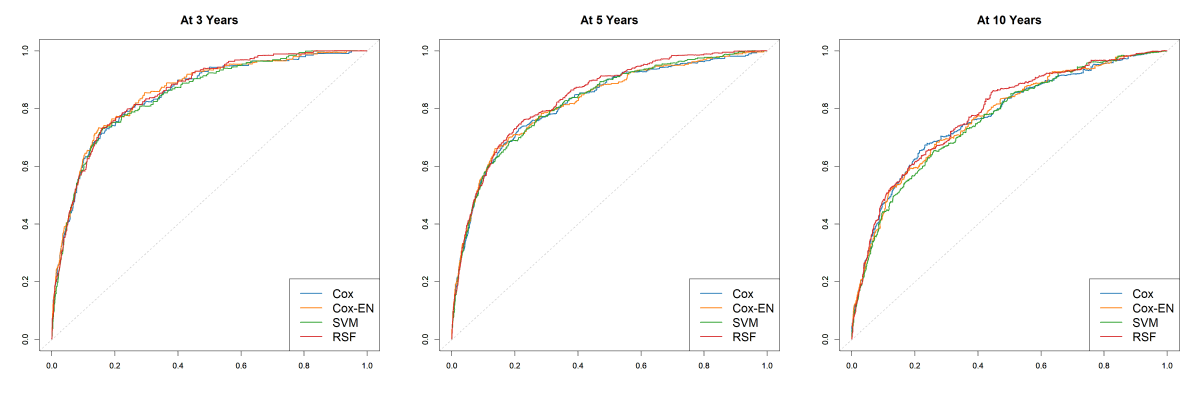
Time-dependent receiver operating characteristic curves of models at 3 years, 5 years, and 10 years. EN: elastic net; RSF: random survival forest; SVM: support vector machine.

**Figure 5:**
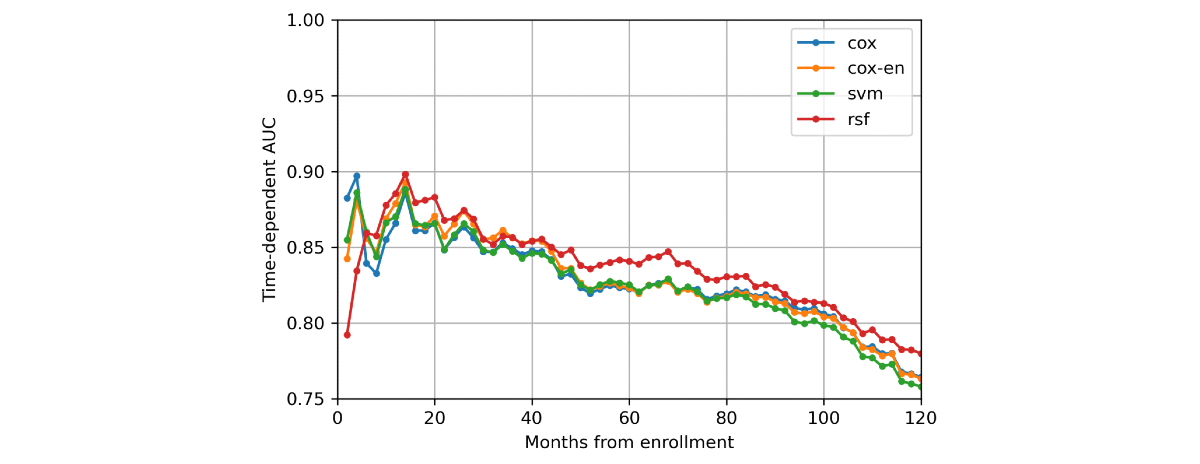
Time-dependent AUC of models over time. AUC: area under the curve; EN: elastic net; RSF: random survival forest; SVM: support vector machine.

**Figure 6:**
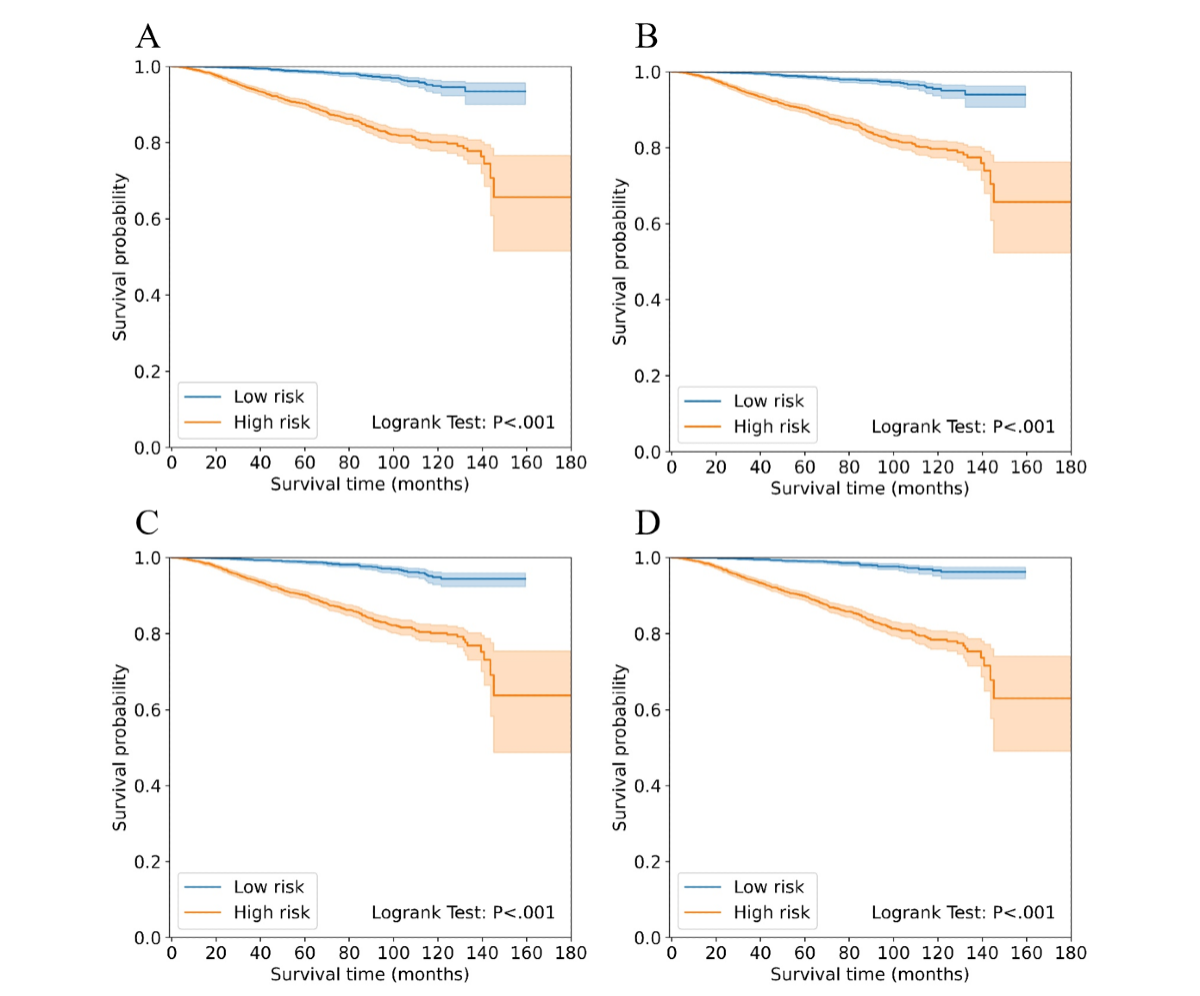
Survival curves of high-risk and low-risk groups divided according to the risk score from (A) Cox, (B) Cox-EN (elastic net), (C) SVM (support vector machine), and (D) RSF (random survival forest).

Evaluation results of the 4 models are shown in Table 1. From the point of view of the C-index, the RSF model slightly and significantly outperformed the Cox-EN model (*P*=.01), the Cox model (*P*=.003), and the SVM model (*P*<.001) on discriminative ability, and no significant difference was found between the discriminative ability of other models. Time-dependent receiver operating characteristic curves of each model at 3 years, 5 years, and 10 years are shown in Figure 4. The time-dependent AUC of each model over time is shown in Figure 5. As shown in Figure 5, the time-dependent AUC of RSF was the highest at most times. Survival curves of the high-risk and low-risk groups divided according to the risk score are shown in Figure 6. The D-index of 7.643 from the RSF model was also the highest, and it can be interpreted as the risk of death in the high-risk group, which is 7.643 times the risk of death in the low-risk group. The 4 models’ 3-year, 5-year, and 10-year Brier scores were all <0.1, suggesting that all models had good calibration.

**Table 1:** Performance of different methods.

Indexes	Cox	Cox-EN^a^	SVM^b^	RSF^c^
C-index^d^ (95% CI)	0.814 (0.794,0.835)	0.816 (0.796,0.836)	0.812 (0.793,0.832)	0.827 (0.809,0.845)
AUC^e^ (3 years)	0.850	0.857	0.847	0.857
AUC (5 years)	0.821	0.822	0.823	0.838
AUC (10 years)	0.770	0.769	0.760	0.781
D-index (95% CI)	7.210 (6.172,8.424)	7.466 (6.383,8.733)	6.522 (5.606,7.583)	7.643 (6.542,8.930)
Brier score (3 years)	0.027	0.027	—^f^	0.027
Brier score (5 years)	0.044	0.045	—	0.045
Brier score (10 years)	0.094	0.093	—	0.093

^a^EN: elastic net.

^b^SVM: support vector machine.

^c^RSF: random survival forest.

^d^C-index: concordance index.

^e^AUC: area under the curve.

^f^Not available. Survival support vector machine can only predict a risk score and not a probability. Therefore, Brier score is not available for survival support vector machine.

### Online Prognostic Prediction Tool

Although the RSF model achieved the best performance among these models, the interpretability and computational efficiency of the RSF model had to be considered at the same time in the deployment of the online prognostic prediction tool. The memory usage of the RSF model was too large for the model to be deployed on a website and have good computational efficiency. The Cox-EN model achieved suboptimal performance in the study and had better interpretability and computational efficiency compared with the RSF model. Therefore, it was selected as a backend for the online prognostic prediction tool [34].

## Discussion

In this paper, we compared the performance of traditional (Cox) and machine learning-based (SVM and RSF) prognostic prediction models for patients diagnosed with breast cancer and found out the RSF model slightly and significantly outperformed the Cox-EN model, the Cox model, and the SVM model on discriminative ability. Compared with Cox, Cox-EN, and SVM, the RSF model had a slightly better performance with a C-index of 0.827 (95% CI 0.809, 0.845) and 3-year, 5-year, and 10-year time-dependent AUC of 0.857, 0.838, and 0.781, respectively. The results in this study were similar to those reported by some previous studies. For example, Liu et al [10] used several methods, including RSF and Cox, to predict breast cancer progression with a sample size of 4575 patients. The results showed that the RSF model achieved better performance with a C-index of 0.814 compared with the Cox model with a C-index of 0.759. Rahman et al [35] showed that RSF (5-year time-dependent AUC 0.839, 95% CI 0.826, 0.849) outperformed Cox (5-year time-dependent AUC 0.823, 95% CI 0.811, 0.833) in the survival prediction of patients with esophageal cancer.

The possible reason for RSF achieving better performance may be that RSF is able to detect and account for higher-order interactions and nonlinear relationships. However, despite the great predictive performance of RSF, there are several shortcomings that limit the wide adoption of RSF. Firstly, the theoretical properties and the inferential procedures of RSF are not well understood. Secondly, RSF creates a “black-box” model that is hard to interpret or visualize [8]. Nonetheless, RSF still has the potential to be used as an effective approach to build prognostic prediction models in the context of survival analysis.

A major advantage of this paper was the large-scale prospective cohort design with a long follow-up time. To the best of our knowledge, this is the study with the largest sample size for breast cancer prognostic prediction modeling based on machine learning in the Chinese population thus far. Even though the study is based on a single institution, the large-scale prospective cohort and long follow-up time make the results valuable and credible.

There are some limitations in this study that should be acknowledged. The main limitation is that this study was performed in a single center in China with no external validation. Therefore, the current results need further multi-institutional validation with larger samples before the prediction models could be used in clinical practice. Another limitation relates to missing data that were imputed, and we could not ascertain the effect of the imputation of missing data on the overall results and subsequent conclusions. Moreover, we chose the randomized search method with 50 parameter settings sampled instead of grid search in the process of tuning the hyperparameters of the RSF due to the limitation of the computational efficiency. This may cause an underestimate of the performance of the RSF model.

In summary, the RSF model slightly outperformed the other models on discriminative ability, revealing the potential of the RSF method to be used as an effective approach to build prognostic prediction models in the context of survival analysis. Our future work will focus on additional external validation of the model using data from multiple centers to verify the extrapolation of our results.

## Appendix

Multimedia Appendix 1 The statistical description of features and the survival curves of patients in the training and test set.

Multimedia Appendix 2 Statistics for missing fields and missing features before and after processing.

Multimedia Appendix 3 Technical implementation details.

Multimedia Appendix 4 Results of univariate survival analysis and multivariate survival analysis.

## Abbreviations

AUC: area under the curve
C-index: concordance index
EN: elastic net
LASSO: least absolute shrinkage and selection operator
RSF: random survival forest
SVM: support vector machine
