# Supplementary material for: The Application and Comparison of Machine Learning Models for the Prediction of Breast Cancer Prognosis: Retrospective Cohort Study
Source: JMIR Med Inform. 2022 Feb 18;10(2):e33440. doi: 10.2196/33440 (PMC8900909; doi:10.2196/33440)
Supplement: Multimedia Appendix 1 [file medinform_v10i2e33440_app1.docx]

**Appendix 1. The statistical description of features and the survival curves of patients in the training and test set**

**Table S1.** The statistical description of features in the training and test set

| Variable | Training^a^ | Test^a^ | *P*-value^b^ | Implication |
| --- | --- | --- | --- | --- |
| Age | 51[44,59] | 51[44,59] | 0.24 | Age at admission |
| BMI | 23.01[21.09,25.00] | 22.89[21.08,25] | 0.18 | Body mass index |
| Diameter | 1.8[1.0,2.5] | 1.8[1,2.5] | 0.17 | Diameter of the tumor (cm) |
| Ln metastasis | 0[0,1] | 0[0,1] | 0.13 | Number of metastatic lymph nodes |
| Menopause |  |  | 0.51 | Menopausal status at admission |
| No | 7126(45.9%) | 3086(46.4%) |  |  |
| Yes | 8397(54.1%) | 3567(53.6%) |  |  |
| Side |  |  | 0.10 | Side of the tumor |
| Right | 7303(47.0%) | 3210(48.2%) |  |  |
| Left | 8220(53.0%) | 3443(51.8%) |  |  |
| Invasive |  |  | 0.19 | Tumor invasion status |
| In situ | 1271(8.2%) | 510(7.7%) |  |  |
| Invasive | 14252(91.8%) | 6143(92.3%) |  |  |
| Multi |  |  | 0.42 | Whether the tumor is multiple or not |
| No | 14880(95.9%) | 6393(96.1%) |  |  |
| Yes | 643(4.1%) | 260(3.9%) |  |  |
| TNM |  |  | 0.90 | TNM staging |
| 0,Ⅰ | 7617(49.1%) | 3239(48.7%) |  |  |
| Ⅱ | 5511(35.5%) | 2362(35.5%) |  |  |
| Ⅲ | 2159(13.9%) | 946(14.2%) |  |  |
| Ⅳ | 236(1.5%) | 106(1.6%) |  |  |
| Ki_67 |  |  | 0.40 | Status of Ki67(>20%,High) |
| Low | 8271(53.3%) | 3504(52.7%) |  |  |
| High | 7252(46.7%) | 3149(47.3%) |  |  |
| ER |  |  | 0.18 | Status of estrogen receptors |
| Negative | 4246(27.4%) | 1878(28.2%) |  |  |
| Positive | 11277(72.6%) | 4775(71.8%) |  |  |
| PR |  |  | 0.72 | Status of progesterone receptors |
| Negative | 5409(34.8%) | 2335(35.1%) |  |  |
| Positive | 10114(65.2%) | 4318(64.9%) |  |  |
| HER2 |  |  | 0.22 | Status of human epidermal growth factor receptor 2 |
| Negative | 11516(74.2%) | 4883(73.4%) |  |  |
| Positive | 4007(25.8%) | 1770(26.6%) |  |  |
| Breast surgery |  |  | 0.83 | Type of Breast surgery |
| Untreated | 153(1.0%) | 60(0.9%) |  |  |
| Breast conserving | 3088(19.9%) | 1332(20.0%) |  |  |
| Mastectomy | 12282(79.1%) | 5261(79.1%) |  |  |
| Axillary surgery |  |  | 0.82 | Type of Axillary surgery |
| Untreated | 520(3.3%) | 214(3.2%) |  |  |
| SLNB | 6155(39.7%) | 2607(39.2%) |  |  |
| ALND | 7472(48.1%) | 3226(48.5%) |  |  |
| SLNB+ALND | 1376(8.9%) | 606(9.1%) |  |  |
| Rebuild surgery |  |  | 0.20 | Type of Rebuild surgery |
| Untreated | 14844(95.6%) | 6355(95.5%) |  |  |
| One-stage reconstruction | 369(2.4%) | 180(2.7%) |  |  |
| Two-stage reconstruction | 310(2.0%) | 118(1.8%) |  |  |
| Adjuvant chemotherapy |  |  | 0.32 | Adjuvant chemotherapy |
| No | 4918(31.7%) | 2063(31.0%) |  |  |
| Yes | 10605(68.3%) | 4590(69.0%) |  |  |
| Targeted therapy |  |  | 0.21 | Targeted therapy |
| No | 13180(84.9%) | 5605(84.2%) |  |  |
| Yes | 2343(15.1%) | 1048(15.8%) |  |  |
| Adjuvant radiotherapy |  |  | 0.18 | Adjuvant radiotherapy |
| No | 9327(60.1%) | 3934(59.1%) |  |  |
| Yes | 6196(39.9%) | 2719(40.9%) |  |  |
| Adjuvant endocrine therapy |  |  | 0.28 | Adjuvant endocrine therapy |
| No | 4748(30.6%) | 2084(31.3%) |  |  |
| Yes | 10775(69.4%) | 4569(68.7%) |  |  |
| Neoadjuvant therapy |  |  | 0.81 | Neoadjuvant therapy |
| No | 13990(90.1%) | 5989(90.0%) |  |  |
| Yes | 1533(9.9%) | 664(10.0%) |  |  |

^a^Categorical variables are described as numbers (percentage), while continuous variables are described as median [1^st^ quartile–3^rd^ quartile].

^b^The difference of categorical variables and continuous variables between training and test set is tested by Chi-square test and Mann–Whitney U test.


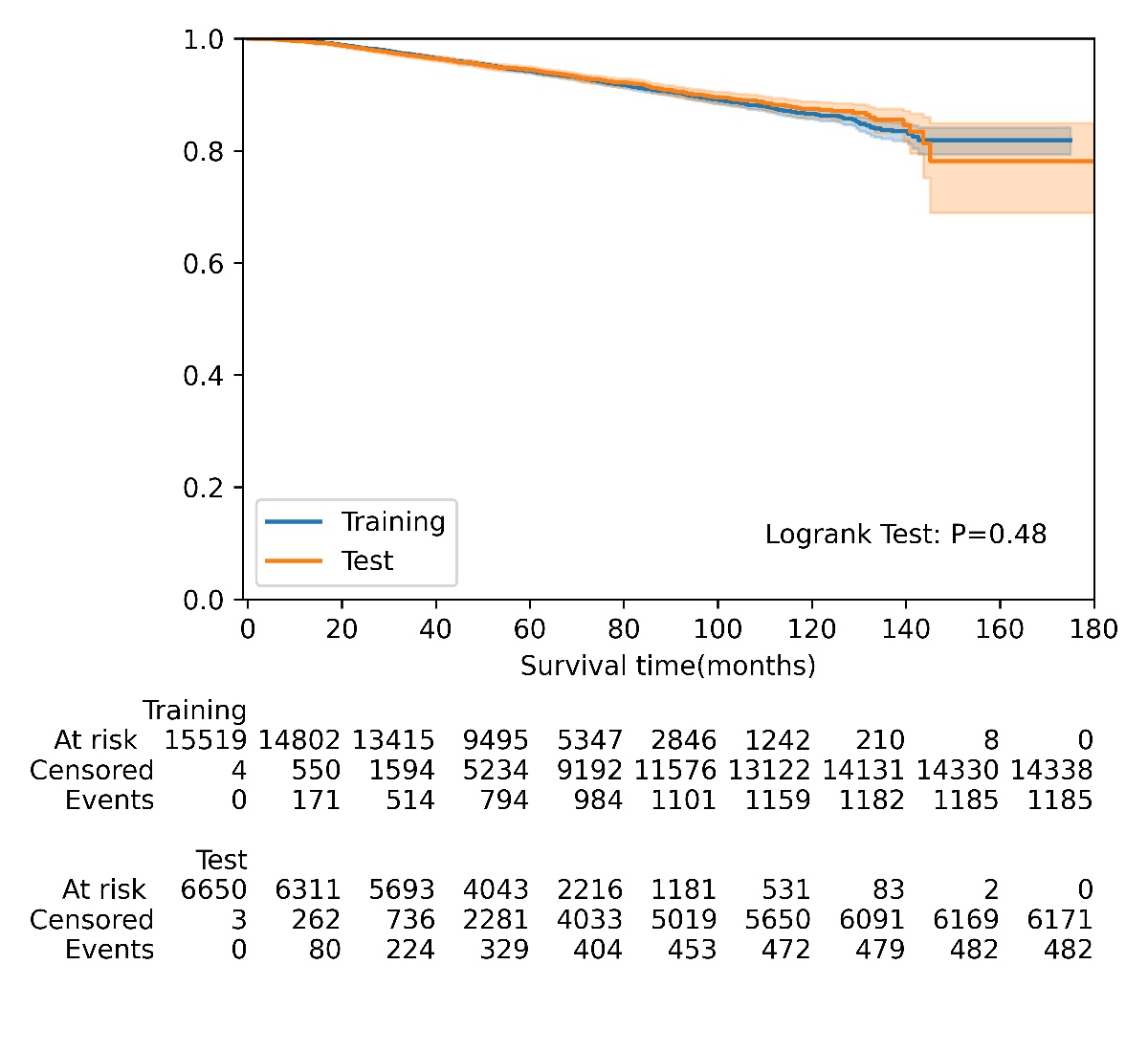


**Figure S1.** Survival curves of the training set and test set.
