# Supplementary material for: The Application and Comparison of Machine Learning Models for the Prediction of Breast Cancer Prognosis: Retrospective Cohort Study
Source: JMIR Med Inform. 2022 Feb 18;10(2):e33440. doi: 10.2196/33440 (PMC8900909; doi:10.2196/33440)
Supplement: Multimedia Appendix 2 [file medinform_v10i2e33440_app2.docx]

| **Appendix 2. Statistics for missing fields and missing features before and after processing**  **Table S1.** Statistics for missing fields | | | | |
| --- | --- | --- | --- | --- |
| No. of Missing features | Excluded patients | Included patients | Log-rank statistics | Log-rank *P*-value |
| ≥12 | 2 | 25265 | 0.000 | >.999 |
| ≥11 | 15 | 25252 | 0.000 | .99 |
| ≥10 | 57 | 25210 | 0.000 | .99 |
| ≥9 | 89 | 25178 | 0.000 | .99 |
| ≥8 | 137 | 25130 | 0.003 | .96 |
| ≥7 | 256 | 25011 | 0.000 | .98 |
| ≥6 | 538 | 24729 | 0.005 | .94 |
| ≥5 | 794 | 24473 | 0.018 | .90 |
| ≥4 | 1583 | 23684 | 0.377 | .54 |
| ≥3 | 3091 | 22176 | 1.930 | .17 |
| ≥2 | 5932 | 19335 | 9.802 | .002 |
| ≥1 | 11384 | 13883 | 11.270 | .001 |

**Table S2.** Statistics for missing features before and after processing

| Variable | Missing (%) Before | Missing (%) After |
| --- | --- | --- |
| age | 0(0.0%) | 0(0.0%) |
| BMI | 749(3.0%) | 540(2.4%) |
| menopause | 0(0.0%) | 0(0.0%) |
| side | 182(0.7%) | 123(0.6%) |
| invasive | 0(0.0%) | 0(0.0%) |
| diameter | 2842(11.3%) | 1419(6.4%) |
| multi | 4(0.0%) | 2(0.0%) |
| ln metastasis | 778(3.1%) | 373(1.7%) |
| TNM | 3043(12.0%) | 1592(7.2%) |
| Ki_67 | 4051(16.0%) | 2622(11.8%) |
| ER | 658(2.6%) | 7(0.0%) |
| PR | 670(2.7%) | 11(0.1%) |
| HER2 | 2708(10.7%) | 1698(7.7%) |
| breast surgery | 0(0.0%) | 0(0.0%) |
| axillary surgery | 0(0.0%) | 0(0.0%) |
| rebuild surgery | 215(0.9%) | 153(0.7%) |
| adjuvant chemotherapy | 1967(7.8%) | 566(2.6%) |
| targeted therapy | 1432(5.7%) | 357(1.6%) |
| adjuvant radiotherapy | 2284(9.0%) | 778(3.5%) |
| adjuvant endocrine therapy | 2295(9.1%) | 893(4.0%) |
| neoadjuvant | 0(0.0%) | 0(0.0%) |
