# Supplementary material for: The Application and Comparison of Machine Learning Models for the Prediction of Breast Cancer Prognosis: Retrospective Cohort Study
Source: JMIR Med Inform. 2022 Feb 18;10(2):e33440. doi: 10.2196/33440 (PMC8900909; doi:10.2196/33440)
Supplement: Multimedia Appendix 3 [file medinform_v10i2e33440_app3.docx]

**Appendix 3. Technical implementation details**

Data were collated using Microsoft Excel and Python libraries pandas (version 1.1.3) and NumPy (version 1.19.2). The machine learning methods, Cox method, and Elastic net are based on the “scikit-survival” package in Python (version 0.14.0). The RSF was used to compute feature importance by eli5 (version 0.11.0) library in Python. C-index was calculated and compared by R libraries Hmisc (version 4.5-0) and survcomp (version 1.40.0). The time-dependent ROC curves were generated by the R library survivalROC (version 1.0.3). The online prognostic prediction tool was built by R libraries shiny (version 1.6.0) and DynNom (version 5.0.1).

Hyper-parameters were tuned by the GridSearch (EN, SVM) and RandomizedSearch (RSF) methods using 5-fold cross-validation in the Python library scikit-learn (version 0.24.2). The elastic net requires the selection of two tuning parameters, r, and α. The parameter r is the relative weight of the l1 and l2 penalty and was set to be 0.9. It is considered to be sufficient to give the l2 penalty only a small weight to improve the stability of the LASSO. Considering the computational efficiency, we chose the RandomizedSearch method with 50 parameter settings sampled in the process of tuning the hyper-parameters of the RSF. The results of tuning hyperparameters of EN, SVM and RSF are shown in **Table S1**.

| **Table S1.** Results of tuning Hyper-parameters | | | | |
| --- | --- | --- | --- | --- |
| **Method** | **parameters** | **range** | **step** | **result** |
| Elastic Net | α | [10^-4^, 10^4^] | 10^0.25a)^ | 10^-1.75^ |
| SVM | α | [2^-12^, 2^13^] | 2^2 a)^ | 2^-10^ |
| RSF | n_estimators | [200, 1000] | 200 | 1000 |
|  | max_depth | [5, 55] | 5 | 10 |
|  | max_features | ['sqrt', 'log2'] | - | log2 |
|  | min_samples_split | [2, 10] | 2 | 4 |
|  | min_samples_leaf | [2, 10] | 2 | 8 |

a) The parameters changed in the form of a geometric sequence.
