# Supplementary material for: The Application and Comparison of Machine Learning Models for the Prediction of Breast Cancer Prognosis: Retrospective Cohort Study
Source: JMIR Med Inform. 2022 Feb 18;10(2):e33440. doi: 10.2196/33440 (PMC8900909; doi:10.2196/33440)
Supplement: Multimedia Appendix 4 [file medinform_v10i2e33440_app4.docx]

**Appendix 4. Results of univariate survival analysis and multivariate survival analysis**

**Table S1.** Results of Univariate Survival Analysis and Multivariate Survival Analysis

| Variable | Group | Univariate Survival Analysis | | | Multivariate Survival Analysis | | |
| --- | --- | --- | --- | --- | --- | --- | --- |
|  |  | P-value | HR | 95%CI of HR | P-value | HR | 95%CI of HR |
| Age |  | <0.001 | 1.031 | (1.027,1.035) | <0.001 | 1.022 | (1.017,1.026) |
| BMI |  | <0.001 | 1.040 | (1.024,1.057) | 0.214 | 0.991 | (0.976,1.005) |
| Side | Left vs Right | 0.695 | 0.981 | (0.891,1.080) | - | - | - |
| Multi | Yes vs No | 0.794 | 1.033 | (0.809,1.319) | - | - | - |
| Menopause | Yes vs No | <0.001 | 2.462 | (2.206,2.747) | <0.001 | 1.401 | (1.250,1.570) |
| Invasive | Yes vs No | <0.001 | 4.842 | (3.394,6.909) | 0.001 | 1.582 | (1.198,2.088) |
| Diameter |  | <0.001 | 1.240 | (1.219,1.261) | <0.001 | 1.109 | (1.083,1.136) |
| Ln metastasis |  | <0.001 | 0.856 | (0.831,0.882) | <0.001 | 1.042 | (1.034,1.050) |
| TNM | Ⅱvs 0,Ⅰ | 0.002 | 0.849 | (0.766,0.940) | <0.001 | 1.382 | (1.217,1.568) |
|  | Ⅲvs 0,Ⅰ | <0.001 | 3.792 | (3.434,4.189) | <0.001 | 2.665 | (2.267,3.134) |
|  | Ⅳvs 0,Ⅰ | <0.001 | 11.564 | (9.864,13.557) | <0.001 | 8.699 | (6.929,10.921) |
| Ki_67 | High vs Low | <0.001 | 2.076 | (1.881,2.292) | <0.001 | 1.462 | (1.321,1.618) |
| ER | Positive vs Negative | <0.001 | 0.526 | (0.477,0.581) | 0.005 | 0.804 | (0.689,0.938) |
| PR | Positive vs Negative | <0.001 | 0.524 | (0.476,0.577) | 0.004 | 0.827 | (0.726,0.943) |
| HER2 | Positive vs Negative | 0.008 | 1.154 | (1.038,1.284) | 0.233 | 0.926 | (0.815,1.051) |
| Breast surgery | Breast conserving vs Untreated | <0.001 | 0.498 | (0.426,0.582) | 0.022 | 0.781 | (0.633,0.965) |
|  | Mastectomy vs Untreated | 0.068 | 1.123 | (0.991,1.273) | <0.001 | 0.698 | (0.572,0.853) |
| Axillary surgery | SLNB vs Untreated | <0.001 | 0.262 | (0.226,0.303) | <0.001 | 0.634 | (0.533,0.753) |
|  | ALND vs Untreated | <0.001 | 2.238 | (2.008,2.493) | 0.463 | 0.942 | (0.803,1.105) |
|  | SLNB+ALND vs Untreated | 0.001 | 0.688 | (0.557,0.849) | 0.018 | 0.763 | (0.609,0.955) |
| Rebuild surgery | One-stage reconstruction vs Untreated | 0.002 | 0.533 | (0.359,0.791) | 0.865 | 0.969 | (0.675,1.391) |
|  | Secondary reconstruction vs Untreated | 0.002 | 0.275 | (0.123,0.614) | 0.469 | 0.804 | (0.445,1.452) |
| Adjuvant chemotherapy | Yes vs No | 0.903 | 1.007 | (0.906,1.118) | <0.001 | 0.746 | (0.662,0.840) |
| Targeted therapy | Yes vs No | 0.673 | 1.030 | (0.898,1.180) | 0.002 | 0.783 | (0.669,0.916) |
| Adjuvant radiotherapy | Yes vs No | <0.001 | 1.388 | (1.261,1.528) | 0.003 | 0.841 | (0.751,0.941) |
| Adjuvant endocrine therapy | Yes vs No | <0.001 | 0.483 | (0.439,0.532) | 0.002 | 0.801 | (0.695,0.925) |
| Neoadjuvant therapy | Yes vs No | <0.001 | 3.489 | (3.124,3.897) | <0.001 | 2.624 | (2.335,2.948) |
